# Service Coverage and Financial Hardship Under Universal Health Coverage: Uneven Associations Across 159 Countries (2000–2023)

**DOI:** 10.3390/ijerph23081069

**Published:** 2026-08-18

**Authors:** Anderson Díaz-Pérez, Wendy Acuña Pérez

**Affiliations:** 1 Centro de Investigaciones en Ciencias de la Vida (CICV), Departamento de Ciencias Sociales y Humanas, Universidad Simón Bolívar, Barranquilla 080002, Colombia; 2 Programa de Enfermería, Facultad de Ciencias de la Salud, Corporación Universitaria Rafael Núñez, Cartagena de Indias 130001, Colombia; wendy.acuna@campusuninunez.edu.co

**Keywords:** universal health coverage, financial protection, health equity, out-of-pocket spending, panel data, global health

## Abstract

**Highlights:**

**Public health relevance—How does this work relate to a public health issue?**

- A harmonized unbalanced panel of 981 country–year observations from 159 countries/economies linked service coverage with financial hardship while retaining wealth- and residence-based strata.
- Global service coverage improved, but financial hardship and subgroup inequalities declined less consistently and remained highly heterogeneous.

**Public health significance—Why is this work of significance to public health?**

- In the primary two-way fixed-effects model, a one-point within-country increase in service coverage was associated with a 0.441-percentage-point reduction in hardship.
- Large wealth- and residence-based gaps persisted despite aggregate progress, showing that national averages can conceal unequal financial exposure.

**Public health implications—What are the key implications or messages for practitioners, policy makers and/or researchers in public health?**

- Universal health coverage monitoring should jointly report service coverage, financial hardship, and subgroup inequality rather than treating any single indicator as sufficient.
- When service coverage rises without parallel reductions in hardship, a financing review should examine pooling, benefit design, medicines coverage, fee exemptions, and point-of-service payments.

**Abstract:**

Universal health coverage requires simultaneous progress in service coverage and financial protection, yet these dimensions are commonly assessed separately. We conducted a secondary ecological panel analysis using official World Health Organization/World Bank indicators. A document-oriented workflow preserved irregular subgroup structures before conversion to an unbalanced country–year panel comprising 981 observations from 159 countries/economies between 2000 and 2023. The primary inferential specification was a two-way fixed-effects model with country and year effects and country-clustered standard errors; a country fixed-effects model with a linear time trend, a random-intercept model, and generalized estimating equations were complementary robustness analyses. We also examined service domains, wealth- and rural-urban inequalities, beta convergence, and exploratory country typologies. Mean service coverage rose from 58.9 in 2000 to 74.0 in 2023, whereas mean financial hardship fell from 24.0% to 17.3%. In the primary model, each one-point increase in service coverage was associated with a 0.441-percentage-point reduction in hardship (95% confidence interval, −0.707 to −0.175; *p* = 0.001). Mean poorest–richest and rural–urban hardship gaps were 53.7 and 12.5 percentage points, respectively. The inverse association was largest in low-income settings, where socioeconomic gradients were also steepest. Service coverage gains should therefore not be interpreted as sufficient evidence of equitable universal health coverage; monitoring systems and financing reforms should jointly target access, financial protection, and distributional gaps.

## 1. Introduction

Universal health coverage (UHC) is a central public health goal because it combines access to needed services with protection from financial hardship. Its two monitoring dimensions, however, can diverge. Countries may expand service contact while households continue to finance care through out-of-pocket (OOP) payments, particularly when revenue collection, risk pooling, strategic purchasing, medicines coverage, or benefit-package design remain incomplete [1,2,3,4,5]. Service coverage should therefore not be assumed to represent financial protection.

Under the revised 2025 Sustainable Development Goal 3.8.2 framework used in this study, overall financial hardship denotes positive out-of-pocket (OOP) health spending exceeding 40% of the household discretionary budget. Impoverishing spending exceeds 100% of that budget, whereas large but non-impoverishing spending lies between the 40% and 100% thresholds [5,6]. The broader literature often uses the term catastrophic health spending for excessive OOP payments, although thresholds differ across definitions. These outcomes are shaped not only by service availability but also by how health systems mobilize, pool, allocate, and govern funds. Evidence from Asia, Africa, and other low- and middle-income settings shows that service expansion may coexist with vulnerability to medicines, chronic care, and hospital costs [7,8,9,10,11].

The distinction became especially visible after the COVID-19 pandemic. Service continuity, workforce capacity, and public financing were simultaneously tested, and disruptions could reduce access while shifting costs onto households. Regional and global evidence consequently links progress toward UHC with resilient primary health care, adequate human resources, and sustained pooled financing [8,9,10,11]. Comparable longitudinal analyses that examine both UHC dimensions across a large set of countries remain limited.

National averages also conceal distributional differences. Financial hardship is rarely evenly distributed by wealth, residence, age, or disability. Poorer households and rural populations may face lower ability to pay, longer travel, greater reliance on informal or private providers, and incomplete entitlement to subsidized services [12,13,14,15,16]. An aggregate improvement can therefore coexist with persistent or widening subgroup gaps.

Existing research provides strong evidence on UHC financing reforms, disease burden, non-communicable disease coverage, maternal and child health, insurance expansion, and oral health inclusion [12,13,14,15,16,17,18,19]. Most studies, however, focus on a single domain, country, region, or financing mechanism. This leaves three connected questions insufficiently resolved: whether within-country growth in service coverage is associated with reduced financial hardship; whether that association differs across geographic and income settings; and whether national progress coexists with wealth- and place-based inequality.

An integrated panel approach is warranted because UHC implementation is path-dependent and depends on governance, institutional sequencing, and accountability [3,19,20,21]. A country with high service coverage may still expose poorer households to substantial OOP spending, while a lower-coverage country may improve financial protection more quickly after financing reform. Analyses must therefore distinguish pooled cross-sectional patterns from within-country change and assess heterogeneity rather than assume a universal pathway.

This study addresses these gaps by harmonizing official service coverage and financial hardship indicators in a country–year panel and preserving irregular subgroup records through a document-oriented non-relational architecture before statistical analysis. It makes four contributions. First, it estimates the within-country association between service coverage and hardship after controlling for time-invariant country characteristics and common year shocks. Second, it evaluates the stability of that association across complementary panel estimators rather than relying on a single specification. Third, it quantifies wealth- and residence-based inequalities that national averages may obscure. Fourth, it develops explicitly exploratory country profiles to support comparative policy learning. We hypothesized that higher service coverage would be associated with lower hardship overall, but that the association would vary across continents and income groups and coexist with persistent within-country inequality [1,2,3,4,5,20,21].

## 2. Materials and Methods

### 2.1. Study Design and Data Sources

We performed a secondary ecological analysis of publicly available aggregate indicators. Service coverage was measured with the World Health Organization/World Bank Universal Health Coverage Service Coverage Index, and financial hardship with the corresponding indicator for hardship due to OOP health spending and its components [6,22,23]. The downloaded service coverage file contained 24,840 raw observations from 207 countries/economies for 2000–2023, including the full index and four domain subindices. The financial hardship file contained 17,634 raw observations from 180 countries/economies for 1985–2024, including overall hardship, component outcomes, wealth quintiles, and urbanization strata.

### 2.2. NoSQL-Oriented Data Architecture

Because the source files combined national records with irregular subgroup dimensions, preprocessing first treated each observation as a document-like unit keyed by country, year, indicator family, and stratifier. This document-oriented non-relational logic preserved combinations of service domain, hardship subtype, wealth quintile, and urbanization without premature flattening or loss of strata.

Three conceptual collections were then derived: service coverage records (full index and domains), hardship records (overall hardship and components), and stratified hardship records (wealth quintiles and urbanization). Only after country–year and subgroup validation were these collections converted to rectangular tables for statistical modeling. The non-relational architecture was therefore a reproducible data engineering step rather than the inferential framework.

### 2.3. Data Cleaning and Analytic Sample

For the overall panel, financial hardship observations were restricted to all household types and total urbanization. The component series—large non-impoverishing burden, impoverishing expenditure, pushed into poverty, and further impoverished—were retained. Service coverage observations included the full index and the four reported domain subindices. After country–year matching and continent assignment, the harmonized unbalanced panel comprised 981 observations from 159 countries/economies between 2000 and 2023. Countries with partial series were retained because the selected estimators can accommodate unbalanced panels.

### 2.4. Variables

The primary exposure was the full service coverage index (0–100). The primary outcome was the percentage of the population experiencing overall financial hardship due to OOP health spending. Secondary outcomes were the four hardship components. The poorest–richest gap was calculated as the difference between the poorest and richest wealth quintiles (Q1–Q5), and the rural–urban gap as the difference between rural and urban hardship. A slope index of inequality (SII) was estimated from quintile-specific hardship values using quintile midpoints as the ranked socioeconomic variable; positive values indicate greater hardship among poorer groups. Countries were also grouped by continent and World Bank income group for stratified interpretation.

### 2.5. External Country Stratification

Countries were stratified using the most recent World Bank analytical income classification available in the Country Analytical History workbook [24]. This external classification was used only for descriptive and stratified analyses, not as an exposure. Two countries/economies lacked a stable match and were excluded only from income-group summaries.

### 2.6. Statistical Analysis

The analytical strategy distinguished one primary inferential specification from complementary estimators. The two-way fixed-effects regression with country and year indicators and country-clustered standard errors was designated as the primary model because the study question concerned within-country change while controlling for time-invariant country characteristics and shocks common to all countries in a given year. In notation, financial hardship_it = β(service coverage_it) + α_i + λ_t + ε_it, where α_i represents country effects and λ_t represents year effects. Thus, β is the average within-country change in hardship associated with a one-point within-country change in service coverage after these adjustments. It remains an associational rather than causal estimand because unmeasured time-varying confounding may persist. Pearson and Spearman correlations were descriptive pooled analyses. A country fixed-effects model with a linear time trend assessed sensitivity to a parsimonious temporal adjustment; a random-intercept mixed model assessed consistency under partial pooling; and generalized estimating equations with exchangeable working correlation provided a population-averaged repeated-measures estimate. These complementary models were used to evaluate robustness, not to select the most favorable coefficient.

The full index was then replaced by its four domains in a mutually adjusted two-way fixed-effects model. Because these domains are related components of one composite construct, their coefficients were interpreted as exploratory conditional associations rather than independent effects. Inequality was summarized using the poorest–richest and rural–urban gaps and the slope index of inequality. Latest country values were compared across continents and income groups; stratified two-way fixed-effects estimates and beta-convergence models were also calculated. For the exploratory typology, five country-level features—latest coverage, latest hardship, annual coverage slope, annual hardship slope, and mean poorest–richest gap—were standardized before k-means clustering. The original workflow fixed k = 4 to balance parsimony, minimum profile size, and policy interpretability; no silhouette, gap statistic, or bootstrap stability result was retained in the archived output. The four-cluster solution is therefore presented as a heuristic summary rather than a uniquely optimal latent classification. All tests were two-sided. Because domain, subgroup, convergence, and clustering analyses were exploratory, *p*-values were interpreted descriptively rather than as confirmatory evidence across multiple hypotheses.

### 2.7. Model Assumptions, Software, Reproducibility, and Ethics

The primary model used country-clustered standard errors, which are robust to heteroscedasticity and allow arbitrary correlation of regression errors within countries. Generalized estimating equations provided a complementary repeated-measures analysis with an explicit exchangeable working correlation. A fixed first-order autoregressive structure was not imposed because observation intervals were irregular and differed across countries. No stand-alone heteroscedasticity or serial-correlation test was used as a model-selection gate, and the archived analytical output did not retain such test statistics; accordingly, the clustered and generalized estimating equation results are treated as inferential safeguards rather than proof that every residual assumption holds. Departure from linearity was evaluated with the reported spline test. These choices reduce sensitivity to common panel-data violations but do not eliminate misspecification or bias from unmeasured time-varying factors. Data processing and analysis were performed in Python 3; the exact minor Python version and package build numbers were not retained in the archived analytical output. The workflow used pandas, NumPy, SciPy, statsmodels, scikit-learn, and matplotlib. The harmonized panel, World Bank crosswalk, reproducible script, and machine-readable outputs are described in the Supplementary Archive (Supplementary File S1–S3). Only public aggregate indicators were used; no identifiable individual-level information was analyzed.

## 3. Results

### 3.1. Global Trends in Service Coverage and Financial Hardship

**Figure 1:**
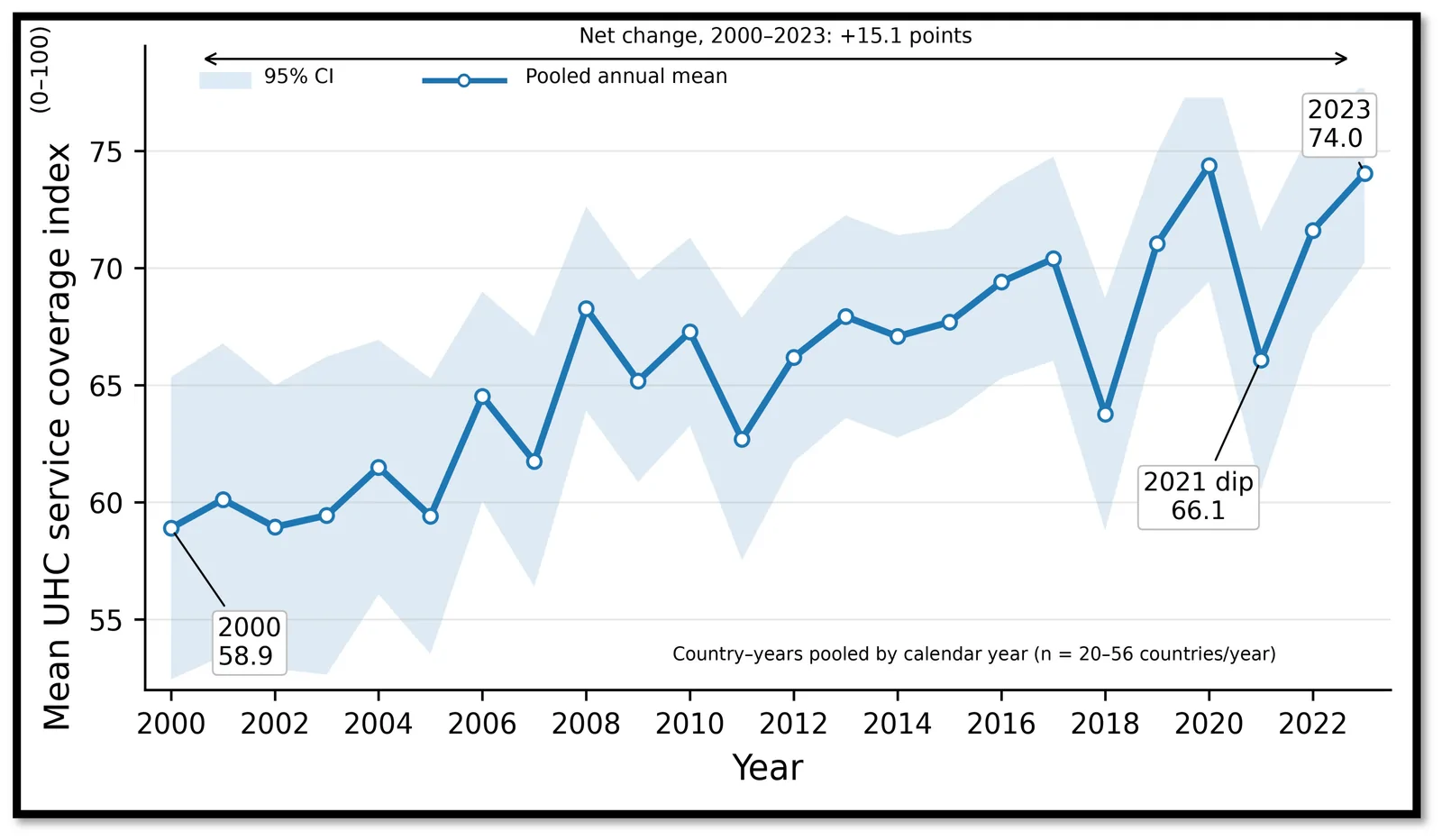
Pooled annual mean Universal Health Coverage Service Coverage Index, 2000–2023. The shaded band represents the 95% confidence interval; annual country counts ranged from 20 to 56.

**Figure 2:**
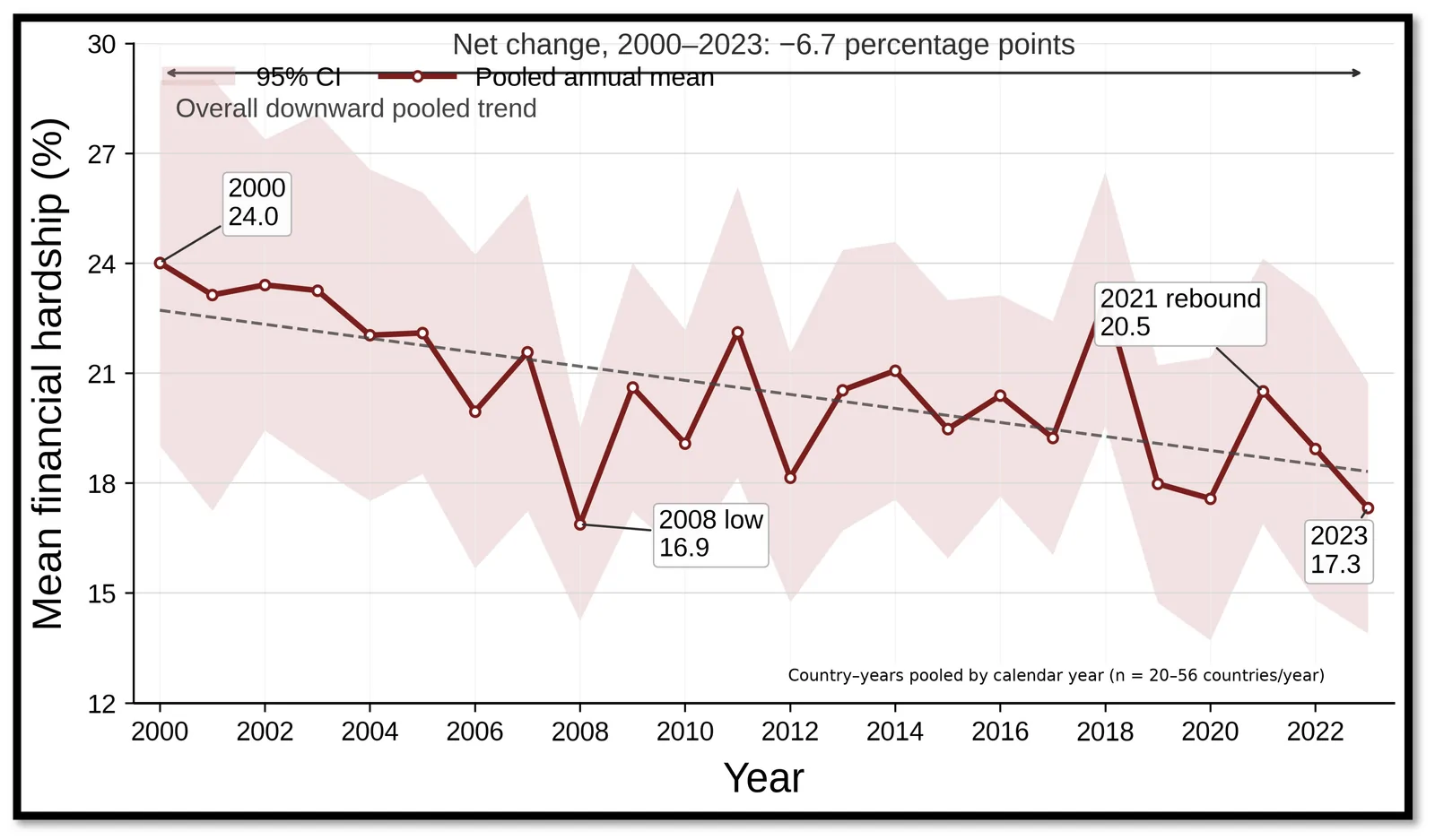
Pooled annual mean financial hardship due to out-of-pocket health spending, 2000–2023. The shaded band represents the 95% confidence interval; annual country counts ranged from 20 to 56. The dashed line represents the fitted pooled linear trend.

The harmonized analytic panel contained 981 country–year observations from 159 countries/economies (Table 1). Mean service coverage across the panel was 65.63 (SD 16.04), and mean overall financial hardship was 20.53% (SD 12.43) (Table 2). Pooled annual means increased from 58.9 in 2000 to 74.0 in 2023 for service coverage and declined from 24.0% to 17.3% for hardship. Because annual country composition varied from 20 to 56 countries, Figure 1 and Figure 2 are descriptive pooled summaries rather than balanced-panel trends.

### 3.2. Main Association Between Service Coverage and Financial Hardship

**Figure 3:**
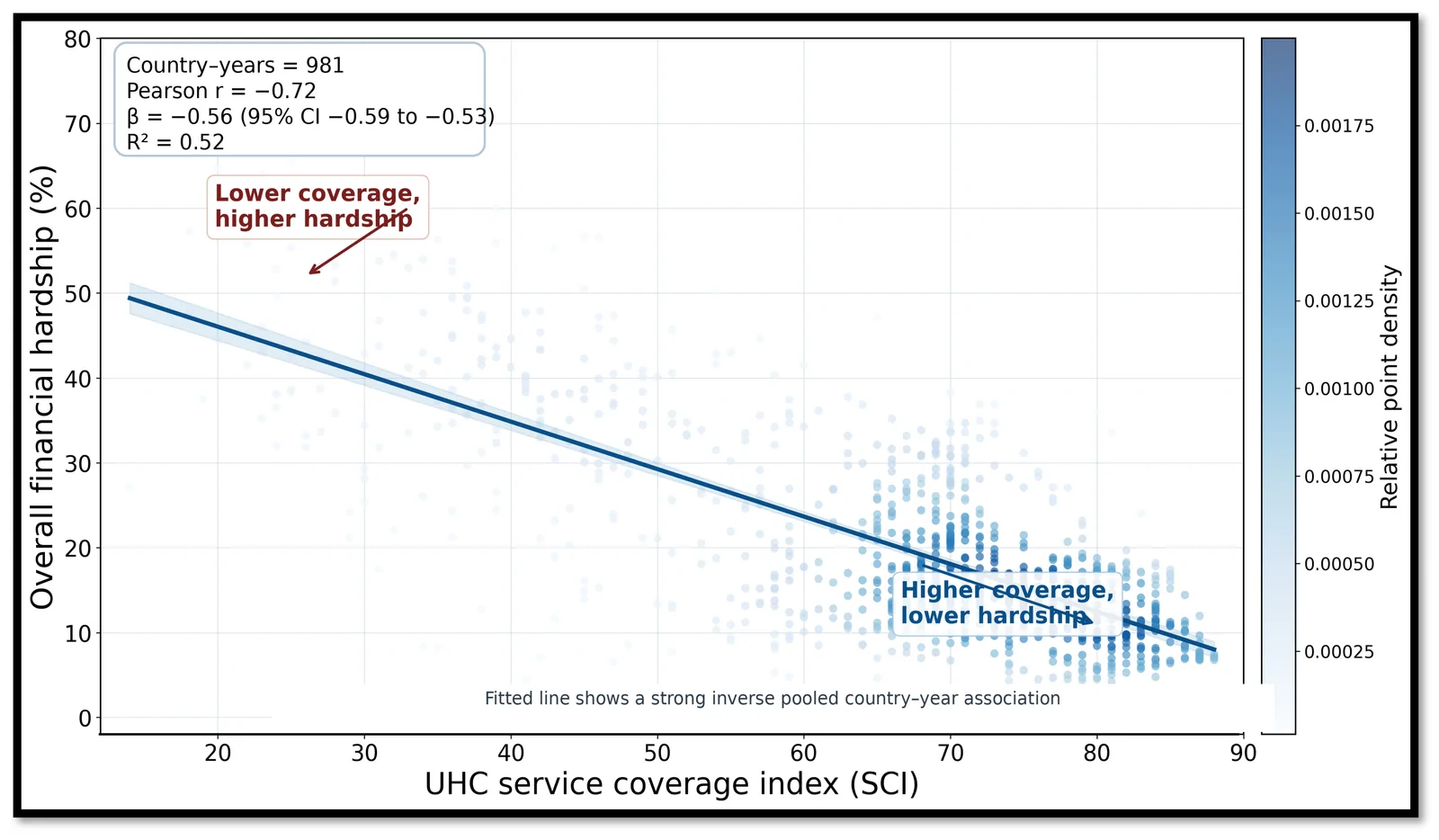
Descriptive pooled association between service coverage and overall financial hardship. Points represent country–year observations; the fitted line and shaded band summarize the unadjusted relationship and its 95% confidence interval. The primary adjusted estimate is reported in Table 3.

The pooled relationship between service coverage and overall financial hardship was strongly inverse (Pearson r = −0.722, *p* < 0.001; Spearman ρ = −0.622, *p* < 0.001). Figure 3 shows this unadjusted country–year pattern. In the primary two-way fixed-effects model, a one-point increase in service coverage was associated with a 0.441-percentage-point reduction in hardship (95% CI, −0.707 to −0.175; *p* = 0.0011). Estimates were similar in the country fixed-effects model with a linear trend (−0.447; 95% CI, −0.691 to −0.203), random-intercept model (−0.496), and generalized estimating equations (−0.521). The spline test provided no strong evidence of nonlinearity (*p* = 0.0618). The consistency of direction and approximate magnitude supports the stability of the association across specifications, but not a causal interpretation (Table 3).

### 3.3. Domain-Specific Associations

In the mutually adjusted domain model, the infectious diseases and non-communicable diseases subindices had the largest inverse conditional associations with hardship: −0.206 percentage points per index point (*p* = 0.0019) and −0.257 percentage points (*p* = 0.0437), respectively. The service-capacity-and-access estimate was also inverse but imprecise (−0.076; *p* = 0.1158). In contrast, the reproductive, maternal, newborn, and child health estimate was positive and imprecise (0.120; *p* = 0.3521). Because the four domains are components of the same index and may be collinear, these coefficients are exploratory and should not be interpreted as isolated causal effects (Table 4).

### 3.4. Inequalities by Wealth Quintile and Urbanization

**Figure 4:**
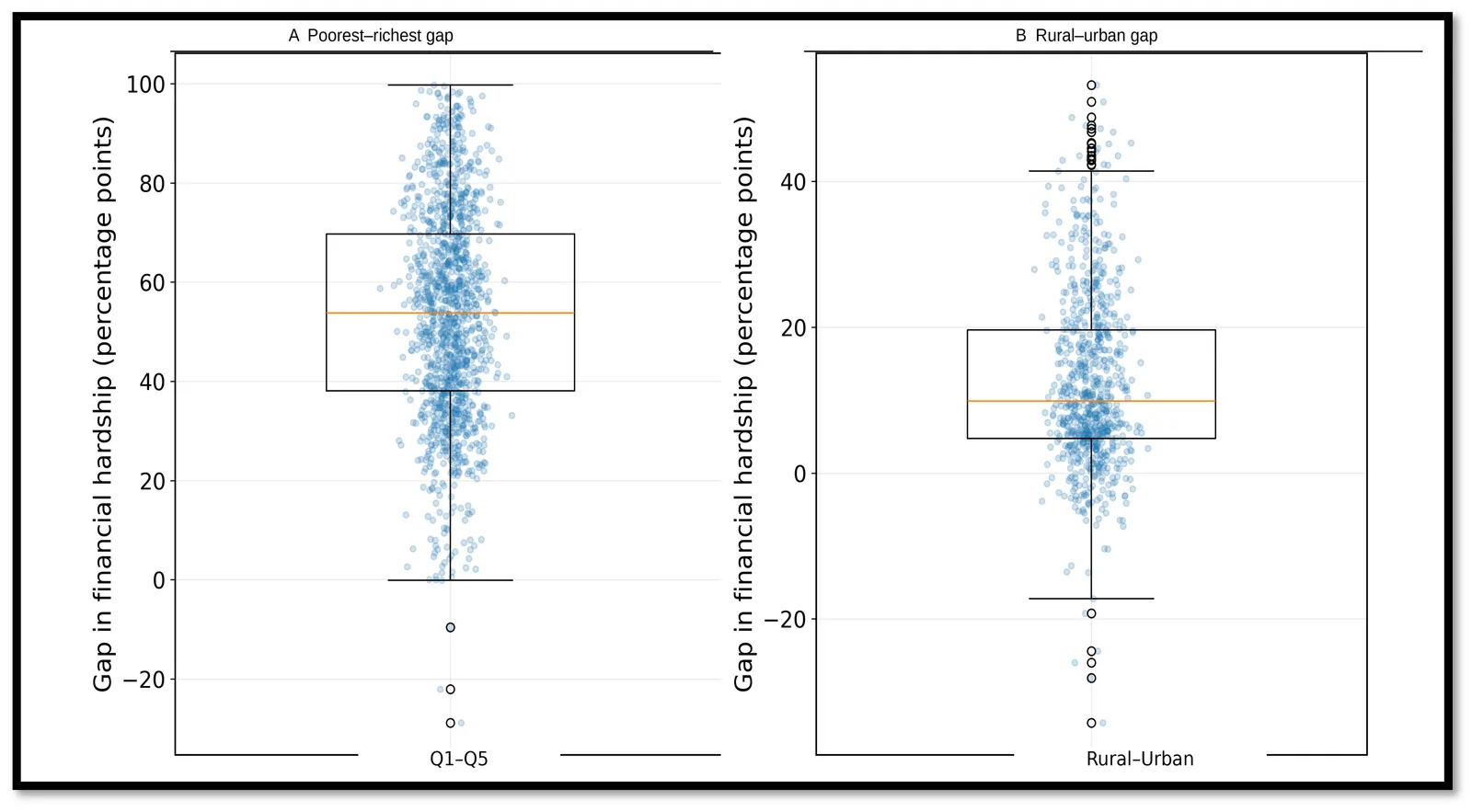
Distribution of wealth- and residence-based financial-hardship gaps across country–years. Blue points represent country–year observations; boxes show the interquartile range, the orange line marks the median, whiskers show the non-outlying range, and open circles denote outliers.

Marked socioeconomic and territorial inequalities were observed wherever disaggregated data were available (Table 5; Figure 4). Across countries and years, the mean poorest–richest hardship gap was 53.73 percentage points, and the mean rural–urban gap was 12.50 percentage points. Positive values predominated, indicating greater hardship among poorer and rural populations, although negative minima show that the direction was reversed in a small number of observations. The latest available SII values were highest in low-income countries (mean 68.85) and in Africa (mean 66.93), and lowest in high-income countries (35.73) and Oceania (20.36) (Table 5, panels A and B).

### 3.5. Cross-Sectional Heterogeneity in Latest Available Values

**Figure 5:**
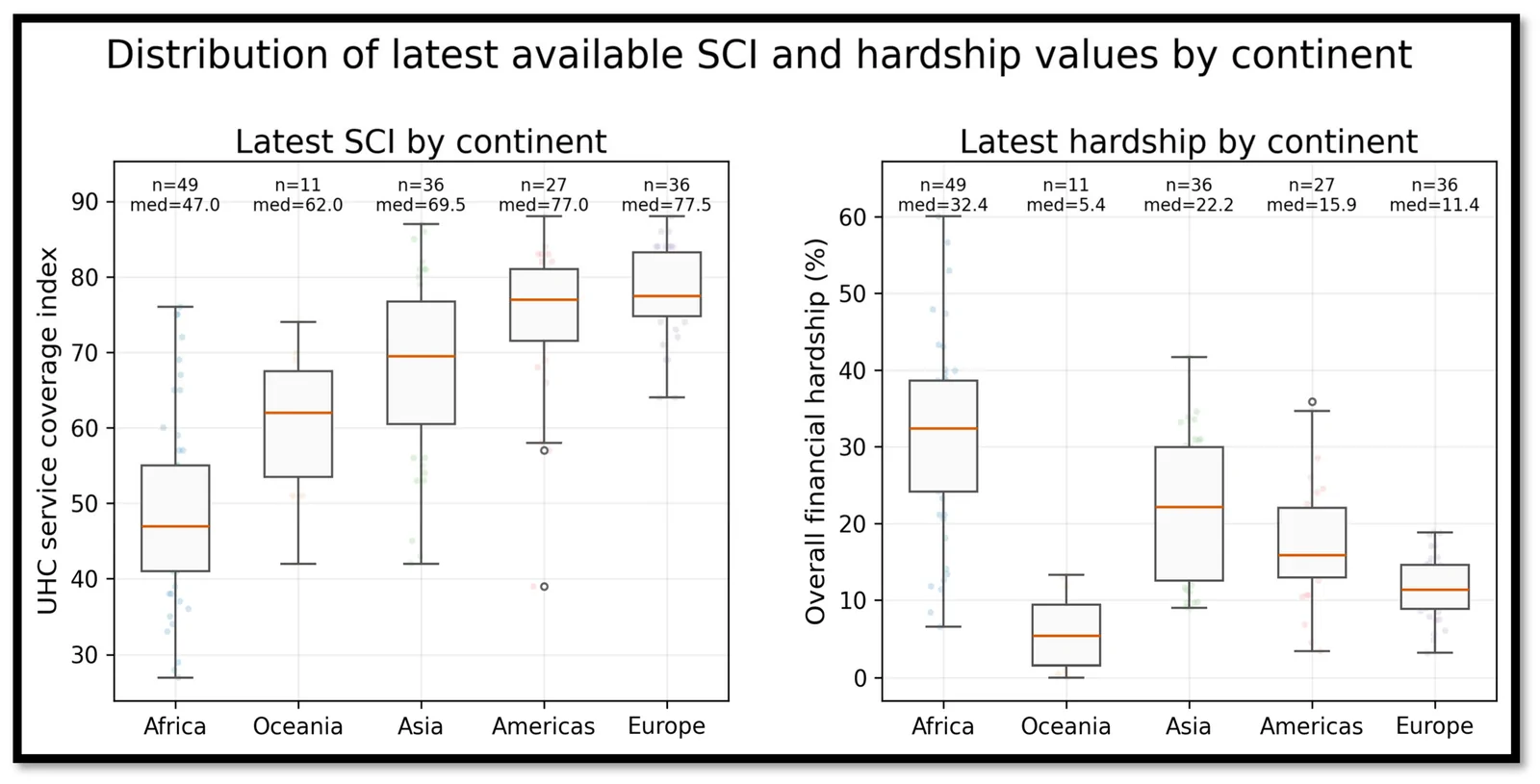
Distribution of latest available service coverage and financial-hardship values by continent. Points represent countries; boxes show the interquartile range, the orange line marks the median, and whiskers show the non-outlying range.

Using each country’s latest available observation, substantial continental differences were evident (Table 6; Figure 5). Europe had the highest mean service coverage (77.94) and comparatively low hardship (11.57%), whereas Africa had the lowest mean coverage (49.06) and the highest hardship (31.29%). Kruskal–Wallis tests indicated between-continent differences for both outcomes (both *p* < 0.001). Across World Bank income groups, mean service coverage rose from 42.21 in low-income settings to 80.05 in high-income settings, while mean hardship fell from 36.36% to 11.49% (Table 7). Because “latest” observations can refer to different calendar years, these comparisons are descriptive.

### 3.6. Stratified Associations, Convergence, and Exploratory Country Typologies

The point estimate for the service coverage–hardship association was largest in Africa (−0.495; 95% CI, −0.779 to −0.211; *p* = 0.0006) and was statistically uncertain in the other continental strata (Table 8). By income group, the largest inverse estimate occurred in low-income countries (−1.002; 95% CI, −1.673 to −0.330; *p* = 0.0035); estimates for high-, upper-middle-, and lower-middle-income groups had confidence intervals that included zero (Table 9). These subgroup estimates indicate heterogeneity but should not be interpreted as statistically significant differences between every pair of strata.

**Figure 6:**
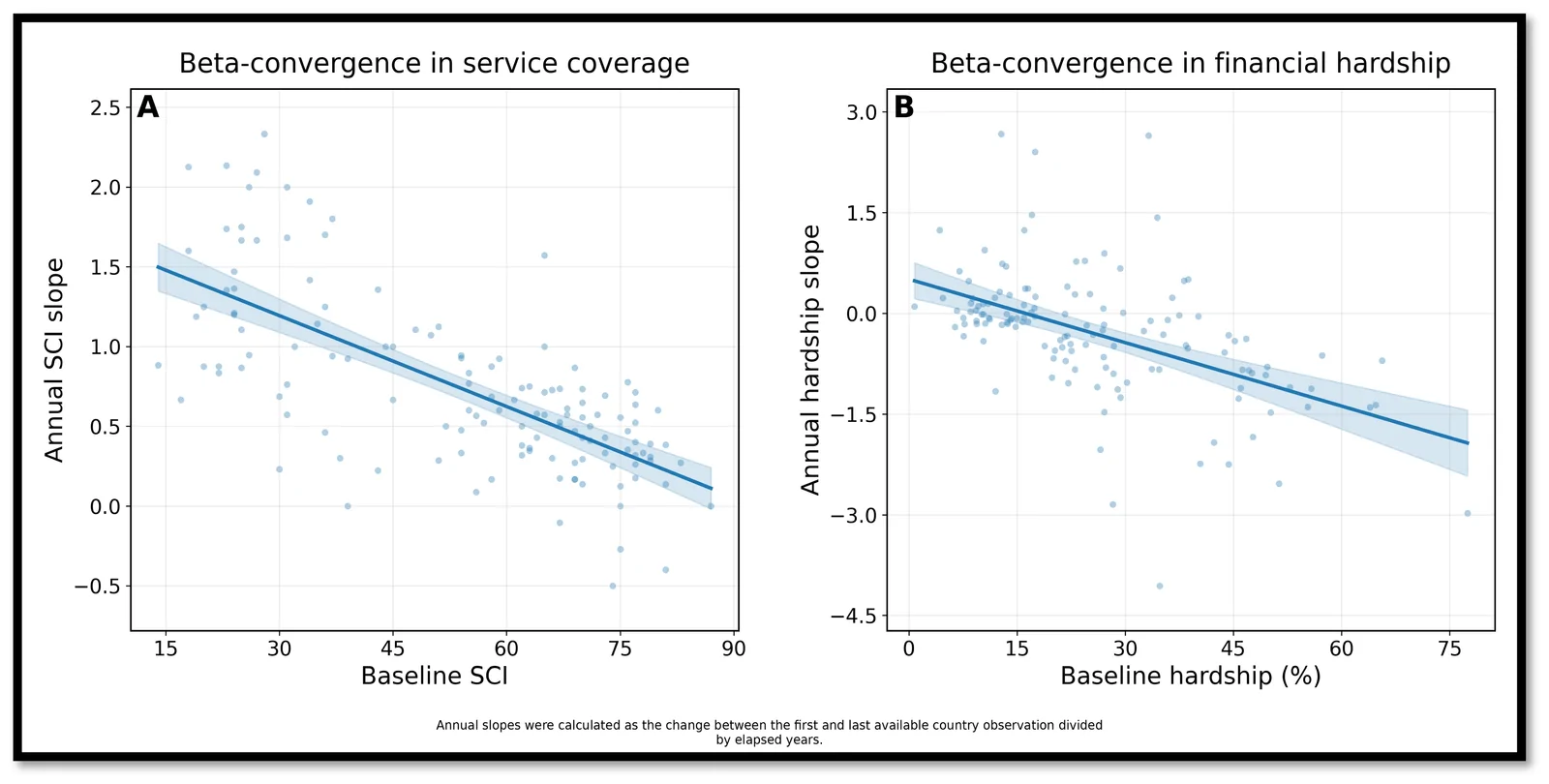
Baseline values and within-country annual slopes for service coverage and financial hardship. Points represent countries; fitted lines summarize beta-convergence patterns, and shaded areas show 95% confidence intervals. The patterns should be interpreted descriptively.

In beta-convergence models, countries with lower baseline service coverage had faster subsequent annual increases (baseline coefficient, −0.019; *p* < 0.001), and countries with higher baseline hardship had larger subsequent declines (baseline coefficient, −0.031; *p* < 0.001) (Table 10; Figure 6). These patterns are descriptive and do not by themselves establish causal convergence.

**Figure 7:**
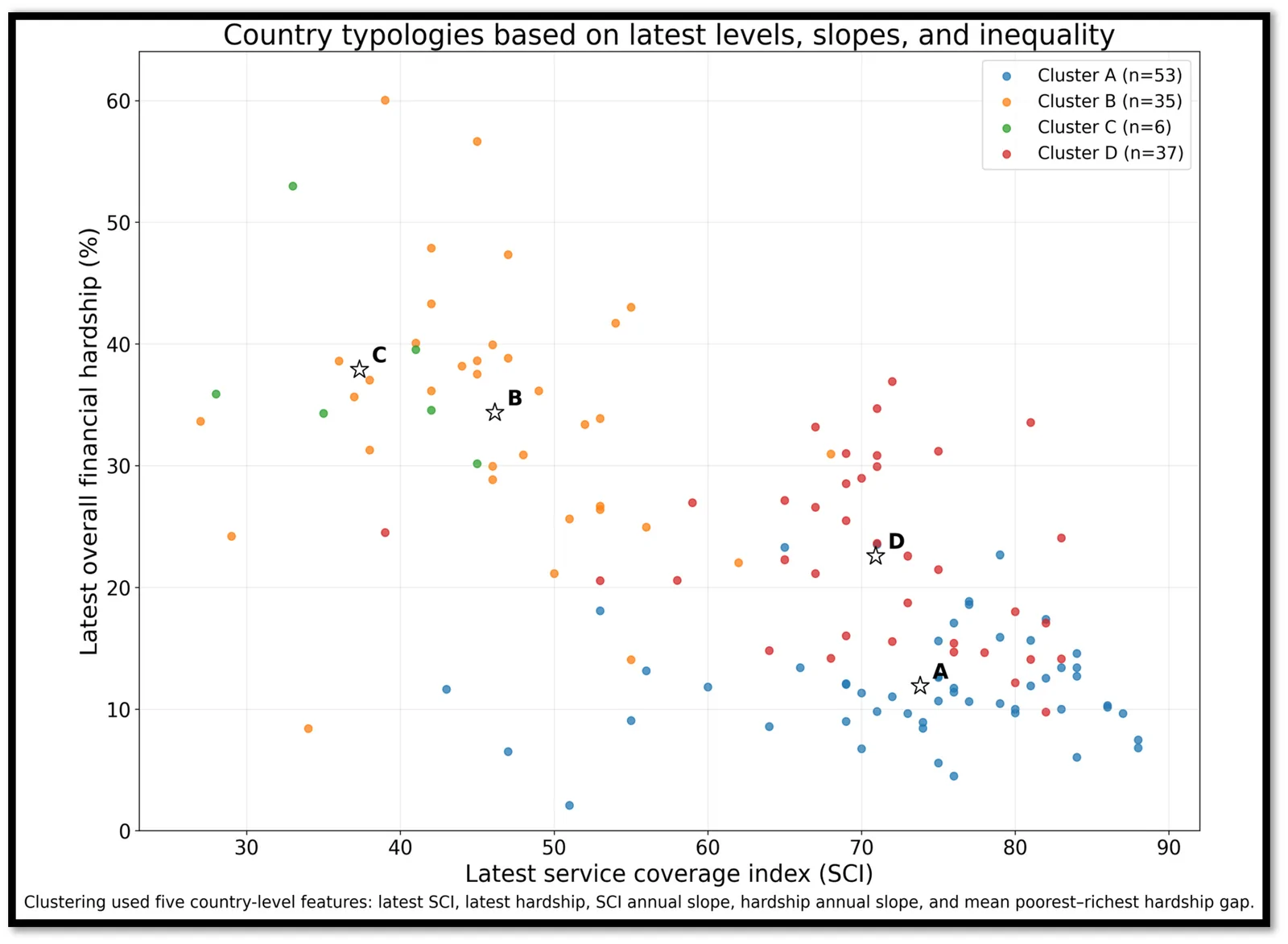
Exploratory country typologies based on latest service coverage, latest financial hardship, within-country slopes, and inequality. Colored points represent countries, and five-pointed stars mark cluster centroids. Cluster labels summarize descriptive profiles rather than causal or validated latent classes.

The prespecified exploratory four-cluster solution summarized distinct policy profiles among 131 countries with complete values for all five clustering features. Cluster A combined relatively high coverage with low hardship; Cluster B combined low coverage, rapid coverage gains, and high inequality; Cluster C was a small group with rising hardship; and Cluster D combined mid-to-high coverage with persistent inequality. The four-cluster solution is a descriptive policy heuristic, not evidence that countries belong to fixed or uniquely identified latent classes (Table 11; Figure 7).

At the country level, the highest latest available service coverage values were observed in the United States and the United Kingdom (both 88), followed by the Republic of Korea (87) and several European high-income systems (Table 12). The highest latest hardship values were concentrated in African countries, led by the Democratic Republic of the Congo (60.03%), Burundi (56.64%), and the Central African Republic (52.97%) (Table 13). Rankings should be interpreted cautiously because countries’ latest observations may refer to different years.

## 4. Discussion

The central finding is not that service coverage and financial protection are unrelated, but that their relationship is incomplete and uneven. The pooled correlation was strongly inverse, and the primary within-country estimate remained negative after country and year effects. Nevertheless, national progress coexisted with large wealth and residence gradients. The service coverage index should therefore be interpreted as one UHC dimension rather than a proxy for financial protection. This conclusion is consistent with regional evidence showing that national UHC gains can coexist with household financial exposure [25,26,27].

The pattern is consistent with health-financing theory. Service availability affects whether care can be obtained, whereas financial protection depends on how care is financed. Coverage expansion is most likely to reduce hardship when revenue is prepaid, risks are pooled broadly, purchasing directs resources toward effective primary care, and benefit design limits payment at the point of use. Conversely, exclusions for medicines or chronic care, copayments, informal payments, and fragmented pools can increase service use without making that use financially safe. This distinction explains why the coverage-hardship coefficient was consistently negative across estimators, yet far too small to offset the observed wealth gradient. Evidence from Thailand, Kenya, Georgia, and East Asia likewise shows that cost sharing, pooling depth, and benefit design mediate whether expanded utilization becomes financially protective [28,29,30,31].

The distributional findings show why national averages are insufficient. Poorer households have less capacity to absorb OOP payments, while rural households may face transport costs, supply shortages, and greater reliance on private or informal providers. The high SII values in Africa and lower-income settings are therefore compatible with both lower fiscal capacity and unequal effective entitlement to subsidized services. Studies from Indonesia, Malaysia, Mexico, and Vietnam likewise show that nominal coverage can coexist with socially patterned financial protection [32,33,34,35,36]. Equity-stratified reporting is thus not an optional supplement to UHC monitoring; it is necessary to identify who is excluded from progress.

The domain-specific findings are hypothesis-generating. Infectious-disease and non-communicable-disease domains had the largest inverse conditional estimates, whereas the reproductive, maternal, newborn, and child health estimate was positive and imprecise. Recurrent medicines and long-term treatment costs may make some domains more directly connected to household spending [27,33,37]. However, the four domains are correlated components of one index, and ecological aggregation can destabilize mutually adjusted coefficients. The results should not be interpreted as evidence that investment in one domain independently causes a specific change in hardship.

The negative baseline coefficients are compatible with beta-convergence, but they may also reflect regression to the mean, different observation intervals, and country-specific shocks. Likewise, the four clusters illustrate heterogeneity rather than proving that countries belong to fixed latent classes. Cluster C contained only six countries, and alternative scaling or cluster numbers could alter membership. The convergence and typology results are therefore best used to generate policy questions and comparative case studies, not to assign permanent labels [37,38].

The policy implication is a differentiated financing agenda. High-income systems should close residual affordability gaps in outpatient medicines, chronic care, dental care, and long-term care and strengthen caps and exemptions for low-income users. Middle-income countries should reduce pool fragmentation, align strategic purchasing with primary care, and make benefits and cost-sharing rules explicit. Low-income countries, particularly in Africa, require pooled tax- and donor-funded primary care, reliable essential-medicines supply, enforceable fee exemptions, and rural workforce investment. Monitoring agencies should also use a discordance trigger: when service coverage improves, but hardship or subgroup gaps do not, the financing architecture—not only service delivery—should undergo formal review, including pooling, purchasing, benefits, medicines coverage, and point-of-service payments [29,30,31,38,39].

Methodologically, the study integrates two major public universal health coverage datasets, preserves irregular subgroup structures through a document-oriented workflow, specifies a primary within-country model, compares complementary estimators, and extends analysis beyond national averages to wealth- and residence-based inequality. The harmonized panel, crosswalk, machine-readable outputs, and reproducible script strengthen transparency and facilitate independent verification.

## 5. Limitations and Future Research

Several limitations constrain interpretation. First, the ecological design cannot identify individual-level mechanisms or establish causality. Second, the unbalanced panel and irregular observation years mean that the pooled annual curves partly reflect changing country composition. Third, survey instruments and estimation procedures underlying official hardship indicators can vary across countries and over time. Fourth, time-varying factors such as public expenditure, insurance reform, inflation, conflict, and benefit design were not modeled directly. Fifth, country-clustered standard errors protect inference against heteroscedasticity and within-country error dependence but do not correct misspecified functional form or omitted time-varying confounding; formal residual test statistics were not retained in the archived output. Sixth, the service domains are conceptually and statistically related; a domain correlation matrix, condition indices, and variance-inflation factors were not available in the archived numerical outputs, so domain coefficients are treated as exploratory. Seventh, k = 4 was selected for a parsimonious policy heuristic rather than demonstrated to be uniquely optimal, and stability across alternative k values and scaling choices was not quantified. Eighth, latest-value rankings combine observations from different calendar years, and multiple exploratory subgroup analyses increase the possibility of chance findings. Finally, current World Bank income groups do not capture historical transitions or all dimensions of fiscal capacity, and two locations lacked a stable match.

Future research should link these indicators with household microdata and time-varying measures of public expenditure, pooling, benefit design, medicines coverage, user fees, inflation, and conflict. Quasi-experimental evaluations of financing reforms could clarify temporal mechanisms. Domain models should report a correlation matrix, condition indices, and variance-inflation factors, while cluster analyses should compare inertia, silhouette width, alternative k values, resampling stability, and out-of-sample persistence. Additional stratification by age, sex, disability, and subnational residence would strengthen equity analysis. These extensions would move the evidence from descriptive global association toward more credible identification of the financing mechanisms that convert service expansion into financial protection.

## 6. Conclusions

Across 159 countries/economies, greater service coverage was associated with lower financial hardship, but the relationship varied across settings and coexisted with large wealth and rural–urban gradients. The primary two-way fixed-effects estimate was directionally consistent with complementary models, yet the ecological design and residual time-varying confounding preclude causal interpretation. Progress in the service coverage index alone is therefore insufficient evidence that universal health coverage has become financially protective or equitable.

Universal health coverage dashboards should report service coverage, hardship components, and subgroup gaps together and should trigger a financing review when these indicators diverge. High-income systems should address residual medicines and chronic-care affordability; middle-income systems should deepen pooling, strategic purchasing, and coherent benefit design; and low-income systems should pair service expansion with pooled public financing, reliable essential-medicines supply, enforceable fee protection, and rural access. Equity-stratified longitudinal monitoring should be used to determine whether reforms are reducing household exposure to financial risk rather than merely increasing contact with services.

## Figures and Tables

**Table 1:** Data sources and analytic sample.

Dataset	Raw Observations	Countries/Economies in Raw File	Time Span	Analytic Filters
UHC service coverage index (SCI)	24,840	207	2000–2023	Full index and four domain subindices
Financial hardship (FH40)	17,634	180	1985–2024	All household types, total urbanization for the overall panel; hardship subcomponents retained
Harmonized analytic panel	981	159	2000–2023	Country–year overlap between service coverage and financial hardship; continent assigned; World Bank income group matched where available

Note. The harmonized panel was produced after country–year matching and the filters described in the Methods section.

**Table 2:** Descriptive statistics for the harmonized panel.

Variable	Mean	SD	Median	IQR	Min	Max	N
SCI full index (0–100)	65.63	16.04	70.0	19.0	14.0	88.0	981
Financial hardship, all (%)	20.53	12.43	16.52	15.61	0.0	77.45	981
Large non-impoverishing OOP burden (%)	3.67	2.47	3.11	3.16	0.0	15.85	959
Impoverishing OOP expenditure (%)	16.85	11.87	12.45	14.49	0.0	73.89	959
Pushed into poverty (%)	1.7	1.19	1.42	1.21	0.0	9.53	885
Further impoverished (%)	15.45	11.75	10.84	13.75	0.2	72.36	885

**Table 3:** Primary and complementary association models linking service coverage to overall financial hardship.

Model	Estimate	95% CI Low	95% CI High	*p*-Value	N	R^2^
Pearson correlation (pooled)	−0.722	—	—	<0.001	981	0.521
Country FE + linear trend	−0.447	−0.691	−0.203	0.0003	981	0.886
Two-way FE (primary)	−0.441	−0.707	−0.175	0.0011	981	0.889
Random-intercept mixed model	−0.496	−0.564	−0.429	<0.001	981	—
GEE (population averaged)	−0.521	−0.599	−0.443	<0.001	981	—
Spline nonlinearity test	—	—	—	0.0618	981	0.890

**Table 4:** Mutually adjusted domain-specific two-way fixed-effects model.

Service Coverage Domain	Estimate (β)	95% CI Low	95% CI High	*p*-Value
RMNCH subindex	0.120	−0.133	0.373	0.3521
Infectious diseases subindex	−0.206	−0.336	−0.076	0.0019
Non-communicable diseases subindex	−0.257	−0.506	−0.007	0.0437
Service capacity and access subindex	−0.076	−0.172	0.019	0.1158

Note. Domain coefficients are exploratory conditional associations. Correlation among component domains may affect coefficient stability; no coefficient should be interpreted as an isolated causal effect.

**Table 5:** Wealth- and residence-based inequality metrics for financial hardship, including (**A**) the latest available slope index of inequality by continent and (**B**) the latest available slope index of inequality by World Bank income group.

Metric	Mean	SD	Median	IQR	Min	Max	N
Poorest–richest hardship gap (Q1–Q5)	53.73	21.40	53.81	31.57	−28.91	99.75	1323
Rural–urban hardship gap	12.50	12.02	9.88	14.90	−34.30	53.23	783
(**A**)							
**Continent**	**Countries**			**Mean SII**		**Median SII**	
Africa	49			66.93		70.59	
Americas	26			45.94		49.71	
Asia	34			56.5		59.17	
Europe	36			36.8		38.81	
Oceania	11			20.36		20.49	
(**B**)							
**Income group**	**Countries**			**Mean SII**		**Median SII**	
High income	44			35.73		35.38	
Upper-middle income	45			46.51		43.38	
Lower-middle income	48			60.13		70.14	
Low income	24			68.85		70.29	

Note. Q1–Q5 is the poorest-quintile value minus the richest-quintile value. Positive rural–urban values indicate greater hardship in rural populations. SII values are complementary measures of the wealth gradient. Country counts reflect locations with sufficient quintile-specific data to compute the SII and therefore differ from totals in Table 6 and Table 7.

**Table 6:** Latest available service coverage and financial hardship by continent.

Continent	Countries	SCI Mean	SCI Median	FH Mean	FH Median
Africa	49	49.06	47.0	31.29	32.44
Oceania	11	60.45	62.0	5.96	5.38
Asia	36	68.08	69.5	21.25	22.16
Americas	27	74.48	77.0	17.51	15.91
Europe	36	77.94	77.5	11.57	11.38

Note. Kruskal–Wallis tests: service coverage, *p* = 1.05 × 10^−17^; financial hardship, *p* = 3.37 × 10^−15^.

**Table 7:** Latest available service coverage and financial hardship by World Bank income group.

Income Group	Countries	SCI Mean	SCI Median	FH Mean	FH Median
High income	41	80.05	81.00	11.49	11.00
Upper-middle income	45	71.64	71.00	16.83	14.64
Lower-middle income	47	56.91	56.00	24.39	26.02
Low income	24	42.21	42.00	36.36	36.02

Note. Kruskal–Wallis tests: service coverage, *p* = 3.79 × 10^−23^; financial hardship, *p* = 9.68 × 10^−14^. Income groups use the most recent World Bank analytical classification. Totals sum to 157 because two countries/economies lacked a stable match.

**Table 8:** Continent-specific service coverage–hardship slopes from stratified two-way fixed-effects models.

Continent	Estimate (β)	95% CI Low	95% CI High	*p*-Value
Africa	−0.495	−0.779	−0.211	0.0006
Americas	−0.223	−0.741	0.294	0.3977
Asia	−0.333	−0.675	0.009	0.0567
Europe	−0.175	−0.689	0.339	0.5047
Oceania	0.062	−0.298	0.422	0.7344

**Table 9:** Income-group-specific service coverage–hardship slopes from stratified two-way fixed-effects models.

Income Group	Estimate (β)	95% CI Low	95% CI High	*p* Value
High income	−0.021	−0.383	0.341	0.9090
Upper-middle income	−0.336	−0.856	0.184	0.2059
Lower-middle income	−0.295	−0.968	0.379	0.3911
Low income	−1.002	−1.673	−0.330	0.0035

**Table 10:** Beta-convergence models for annual within-country change.

Outcome	Baseline Coefficient	95% CI Low	95% CI High	*p* Value	R^2^
SCI annual slope	−0.019	−0.022	−0.016	<0.001	0.487
FH annual slope	−0.031	−0.041	−0.022	<0.001	0.260

**Table 11:** Exploratory four-cluster country typology.

Cluster	Profile	Countries	SCI (Latest)	Hardship (Latest)	SCI Annual Slope	FH Annual Slope	Mean Q1–Q5 Gap
A	High coverage; low hardship	53	73.81	11.90	0.54	−0.08	36.40
B	Low coverage; rapid gains; high inequality	35	46.14	34.37	1.37	−0.91	65.65
C	Fragile; rising hardship	6	37.33	37.90	0.63	1.79	33.91
D	Mid-to-high coverage; persistent inequality	37	70.92	22.57	0.48	−0.40	67.87

Note. Clustering used standardized latest coverage, latest hardship, annual coverage slope, annual hardship slope, and mean poorest–richest gap. The 131-country complete-case sample and the small size of Cluster C (n = 6) warrant cautious interpretation.

**Table 12:** Countries with the highest latest available service coverage values.

Country	Continent	Service Coverage (Latest)	Hardship (Latest)
United States	Americas	88.0	6.81
United Kingdom	Europe	88.0	7.47
Republic of Korea	Asia	87.0	9.65
Finland	Europe	86.0	10.15
Switzerland	Europe	86.0	10.31
Japan	Asia	86.0	11.0
Israel	Asia	85.0	16.57
Netherlands	Europe	84.0	3.17
Germany	Europe	84.0	4.8
Slovenia	Europe	84.0	6.05

**Table 13:** Countries with the highest latest available financial hardship values.

Country	Continent	Service Coverage (Latest)	Hardship (Latest)
Democratic Republic of the Congo	Africa	39.0	60.03
Burundi	Africa	45.0	56.64
Central African Republic	Africa	33.0	52.97
Sierra Leone	Africa	42.0	47.89
Nigeria	Africa	47.0	47.35
Liberia	Africa	42.0	43.29
Malawi	Africa	55.0	43.02
Bangladesh	Asia	54.0	41.7
Mali	Africa	41.0	40.07
Sudan	Africa	46.0	39.92

## Data Availability

The original service coverage and financial hardship indicators are publicly available from the WHO/World Bank monitoring ecosystem [6,22,23]. The harmonized analytic panel, income-group crosswalk, reproducible script, and machine-readable outputs are contained in the Supplementary Archive or are available from the corresponding author upon reasonable request.

## Supplementary Materials

The following supporting information can be downloaded at: https://www.mdpi.com/article/10.3390/ijerph23081069/s1, File S1: Reproducible Analysis; File S2: Harmonized Data; File S3: Machine Readable Data Underlying Main Tables. The supplementary archive contains the reproducible Python analysis script, the harmonized country–year panel and income-group crosswalk, and machine-readable outputs.

## Abbreviations

SII	slope index of inequality
CICV	Centro de Investigaciones en Ciencias de la Vida
OOP	out-of-pocket
FH40	financial hardship due to OOP health spending
95% CI	95% confidence interval
JSON	JavaScript Object Notation
ORCID	Open Researcher and Contributor ID
Q1	poorest wealth quintile
RMNCH	reproductive, maternal, newborn, and child health
SD	standard deviation
UHC	universal health coverage
CI	confidence interval
CSV	comma-separated values
FH	financial hardship
GEE	generalized estimating equations
WB	World Bank
NoSQL	non-relational, document-oriented data architecture
PNG	Portable Network Graphics
Q5	richest wealth quintile
SCI	service coverage index
SDG	Sustainable Development Goal
WHO	World Health Organization
